# Developing an Index to Measure Structural Racism: Methodological Process, Challenges, and Considerations

**DOI:** 10.3390/ijerph23020200

**Published:** 2026-02-03

**Authors:** Christopher M. Amissah, Alisha A. Crump, Yu-Hua Fu, Sheela Khadka, Jennifer Contreras, Salene M. W. Jones, Bryce B. Reeve, Ester Villalonga-Olives

**Affiliations:** 1 Department of Practice, Sciences, and Health Outcomes Research, School of Pharmacy, University of Maryland, Baltimore, MD 21201, USAjcontreras@umaryland.edu (J.C.); 2 Graduate Program in Psychometrics, Department of Psychology, Morgan State University, Baltimore, MD 21251, USA; 3 School of Global Public Health, New York University, New York, NY 10003, USA; 4 Hutchinson Institute for Cancer Outcomes Research (HICOR), Public Health Sciences Division, Fred Hutch, Seattle, WA 98109, USA; 5 Department of Population Health Sciences, School of Medicine, Duke University, Durham, NC 27710, USA; bryce.reeve@duke.edu

**Keywords:** index development, ecological-level index, structural racism, health equity, health disparities, Delphi process, data challenges, measure development, methodological challenges, psychometrics

## Abstract

**Highlights:**

**Public health relevance—How does this work relate to a public health issue?**

- Examines methodological challenges in developing ecological measures of structural racism affecting Black and Hispanic populations.
- Provides methodological guidance for developing ecological measures of structural racism to support research on social determinants of health.

**Public health significance—Why is this work of significance to public health?**

- Offers a replicable, consensus-driven approach for identifying indicators of structural racism using public datasets.
- Presents a five-step approach for extracting and harmonizing geographic-level datasets for structural racism index development.

**Public health implications—What are the key implications or messages for practitioners, policy makers and/or researchers in public health?**

- Strengthens researchers’ capacity to develop measures to assess structural determinants of health disparities and inform equity-focused interventions.
- Highlights the need for improved public data infrastructure to support equity monitoring.

**Abstract:**

Access to valid and reliable measures of structural racism is essential for addressing health inequities, yet few validated ecological-level indices exist for assessing structural racism affecting Black and Hispanic populations in the United States. Guided by the National Institute on Minority Health and Health Disparities framework, our interdisciplinary team undertook the development of an ecological-level structural racism index. In the process, we encountered substantive methodological and data-related challenges that warrant explicit documentation. This paper describes the methodological process used to identify and select indicators of structural racism, including a modified Delphi consensus process involving social epidemiologists, health inequality researchers, community members, economic inequality specialists, and psychometricians. We outline a five-step approach for extracting and harmonizing geographic-level data from publicly available sources and discuss key challenges encountered, including limited availability of granular geographic data, insufficient data documentation guidelines, inconsistent reporting frequencies, and difficulties in adapting publicly available datasets for structural racism measurement. Rather than presenting a finalized index, this paper serves as a methodological guide and cautionary account for researchers seeking to develop ecological measures of structural racism, emphasizing the importance of transparency, adaptability, and rigorous data selection in advancing public health equity research.

## 1. Introduction

Structural racism represents the totality of ways in which multiple systems and institutions interact to produce and sustain racist policies, practices, and beliefs about people in a marginalized group [1]. Structural racism, deeply embedded in societal institutions, is a critical determinant of health inequities and social disparities [2]. The development of measures of structural racism has become increasingly important in epidemiological and public health research [3]. Recent advancements in the literature have shown a significant shift from single-domain indicators of structural racism, such as the use of redlining or incarceration rates, to cumulative multidimensional ecological indices of structural racism [4,5,6,7]. These indices provide a quantitative framework to assess the multifaceted nature of structural racism, enabling researchers to examine its impact on various health outcomes and social inequalities across different populations and geographic areas. For instance, Dougherty et al. developed a 5-domain index that captures county-level structural racism in education, housing, employment, criminal justice, and healthcare, which they applied to body mass index disparities in Black populations [6]. Similarly, Chantarat et al. introduced a comprehensive measure of structural racism in Public Use Microdata Areas (PUMAs), incorporating indicators for Black-White residential segregation and inequities in education, employment, income, and home ownership [5]. Dyer et al. also developed a structural racism index by organizing publicly available data on 42 variables at the census tract level, covering nine domains affected by structural racism, including built environment, criminal justice, education, employment, housing, income and poverty, social cohesion, transportation, and wealth [7]. Additionally, Brown and Homan created a state-level structural racism measure, comprising five domains: the economy, education, politics, the criminal-legal system, and residential segregation [4]. These multidimensional approaches provide a more nuanced understanding of the interactive effects of structural racism across multiple institutions, offering valuable insights for policymakers and researchers alike.

While recent studies have made substantial progress in developing multidimensional ecological-level indices of structural racism, most existing measures were originally conceptualized to capture Black–White structural inequities and are typically applied at a single level of analysis. Several studies have since extended these indices to examine associations with health outcomes across racial and ethnic groups, including Hispanic/Latino populations. For example, recent ecological analyses have applied a census tract–level Structural Racism Effect Index across neighborhoods to examine cardiovascular risk factors and disease prevalence, including comparisons involving Hispanic ethnicity [7,8]. However, these applications rely on indicator sets originally developed for Black–White comparisons and do not explicitly incorporate structural processes that uniquely shape Hispanic/Latino experiences, such as language-based exclusion, immigration enforcement environments, or access to culturally responsive institutions. As a result, important dimensions of structural racism affecting Hispanic/Latino communities may be underrepresented, underscoring the need for measurement frameworks intentionally designed to assess structural racism affecting both Black and Hispanic/Latino populations while supporting comparability across groups.

Our work is motivated by the need to extend these existing efforts in three key ways. First, our long-term goal is to develop a multilevel and multidimensional measure of structural racism that integrates ecological-level indicators with complementary individual-level measures. Existing indices largely focus on a single level of analysis, which limits the ability to examine how structural conditions interact with individual experiences. Second, building on prior indices, our approach incorporates a broader and more comprehensive set of indicators spanning multiple structural domains than most existing indices. Expanding the indicator set required explicit, interdisciplinary consensus-building and careful data harmonization, both of which raise methodological challenges that have received limited attention in the literature. Third, while much of the existing measurement work has focused primarily on structural racism affecting Black populations, our framework is explicitly designed to assess structural racism affecting both Black and Hispanic populations. This requires additional conceptual and methodological considerations, including the selection of indicators that are relevant across groups while remaining sensitive to group-specific structural processes. The present paper focuses on documenting the methodological processes and challenges involved in laying the groundwork for a comprehensive, multilevel measurement framework, rather than presenting a finalized index.

To develop the multilevel measure, we worked on the development of an ecological-level index designed for use among Black and Hispanic/Latino populations living in the United States. While racism affects both Black and Hispanic/Latino populations in profound and lasting ways [9,10,11,12], there is limited understanding of how the nature and impact of structural racism may differ between these two groups. Much of the existing research on structural racism has focused predominantly on the experiences of Black Americans, resulting in a conceptual and empirical gap in our knowledge about how systemic discrimination operates against Hispanic/Latino communities. This lack of attention is especially concerning given that Hispanics/Latinos now represent the largest minority group in the United States, with diverse backgrounds and experiences shaped by ethnicity, immigration status, language, acculturation, and national origin [13,14]. Despite this demographic significance, few measures have been developed to specifically capture the forms of exclusion, marginalization, and inequality faced by Hispanic/Latino individuals [10,15]. Addressing this gap is essential for developing an inclusive and accurate understanding of structural racism and its consequences for population health and quality of life.

To guide the development process, we involved an expert panel and used a modified Delphi process to reach consensus on the key components and dimensions necessary for constructing an effective measure for the specified populations. We purposively highlight the challenges encountered during the development process and discuss the strategies employed to address these challenges. The goal of presenting these challenges and solutions is for researchers to gain insights that can inform the use of our index and the development of similar indices in future research. By transparently outlining both obstacles and methodological decisions, we aim to contribute to a growing body of work that critically engages with the measurement of structural racism and promotes methodological rigor and innovation in this area.

As part of our goal to develop a multilevel model, we are also working on an individual-level measure to complement the ecological-level index of structural racism, which will be presented in future work. This multilevel approach aims to capture both personal experiences and broader structural forces, ultimately providing a more comprehensive and accurate understanding of how structural racism operates.

## 2. Materials and Methods

### 2.1. The NIMHD Framework

**Figure 1:**
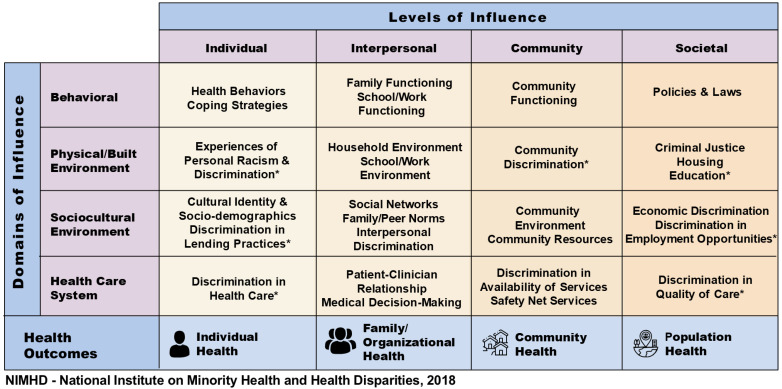
An adapted NIMHD model for the development of the structural racism index. Note: The biological domain from the original model [16] was excluded. Domains marked with an asterisk (*) represent those adapted for the current study. Structural racism indicators were mapped to the Behavioral, Physical/Built Environment, Sociocultural Environment, and Health Care System domains across all four levels of influence (Individual, Interpersonal, Community, and Societal).

We adapted the U.S. National Institute on Minority Health and Health Disparities (NIMHD) Research Framework to guide the development of our structural racism index [16,17]. This conceptual framework underscores the role of structural mechanisms in creating and sustaining health inequities across multiple levels of influence, including the individual, interpersonal, community, and societal levels. It delineates key domains through which these mechanisms operate, such as biological, behavioral, physical/built environment, sociocultural environment, and the healthcare system. Applying this multidimensional and multilevel framework, we conceptualized structural racism as a systemically embedded force that operates across domains and levels of influence. While the original framework encompasses the biological domain, we excluded it from our study to focus on the social, environmental, and systemic factors most pertinent to the measurement of structural racism in our study (see Figure 1). The biological domain will be addressed in future research. This targeted application of the NIMHD Research Framework allowed us to align our index with the social and structural processes that drive racial and ethnic health inequities.

### 2.2. Three-Phase Process of Ecological-Level Index Development

**Figure 2:**
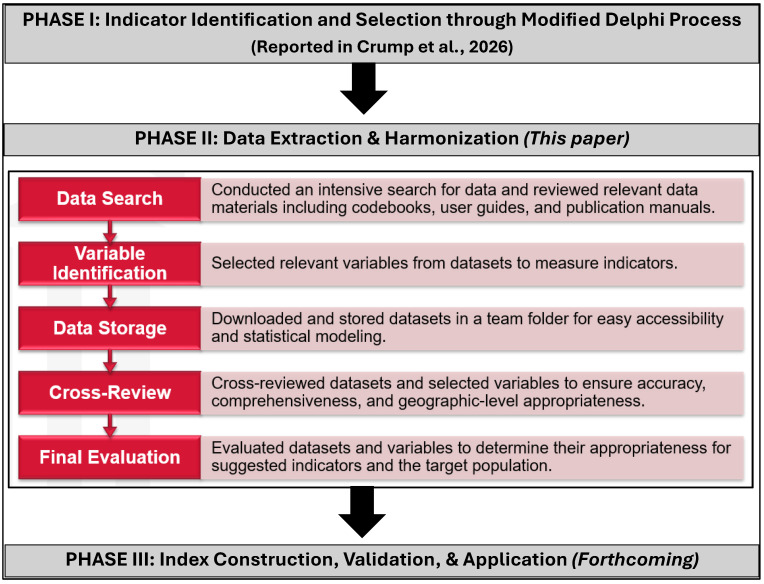
Flowchart of the three-phase process for developing an ecological-level structural racism index [18].

Our approach to developing the ecological-level structural racism index is structured across three phases. Phase I involved selecting indicators of structural racism through a modified Delphi process with input from experts across various disciplines, a process previously reported [18]. Phase II focused on extracting and harmonizing geographic-level data through a five-step approach: (1) conducting a data search and reviewing relevant materials, (2) identifying variables to measure key indicators, (3) organizing and storing datasets, (4) cross-reviewing datasets for accuracy, and (5) evaluating datasets for their relevance to the proposed indicators and target populations. This paper specifically reports on Phase II, with particular emphasis on the methodological challenges encountered and the strategies used to address them during data extraction and harmonization. Phase III is forthcoming and will involve constructing, validating, and applying the finalized index to health outcomes. Figure 2 provides an overview of the three-phase process for developing the ecological-level structural racism index and highlights the five-step approach for data extraction and harmonization in the current phase.

### 2.3. Delphi Process

Our team is composed of a content development team, including social epidemiologists with expertise in health disparities (*N* = 4), and a measurement team composed of psychometricians (*N* = 2). The expert panel included health equity researchers specializing in structural racism and its effects on health (*N* = 5), community organization leaders who provided insight into the lived experiences of Black and Hispanic/Latino communities (*N* = 3), and social stratification researchers with knowledge of the financial systems that disproportionately disadvantage racial minorities (*N* = 2). Further details regarding panel recruitment, community representation, compensation, and methodological adaptations to the Delphi process are reported separately [18]. The overview provided here is intended to document the logic and transparency of indicator selection relevant to index development, rather than to provide a comprehensive evaluation of the Delphi methodology itself.

To reach consensus on the selection of indicators for the structural racism index, we employed a modified Delphi process [19,20]. The content development team began by pre-selecting an initial set of ecological-level indicators through a comprehensive review of the literature and established indices, such as those developed by Dougherty et al. and Dyer et al. [6,7]. Through three iterative rounds, the research team assessed each indicator’s relevance and alignment with the NIMHD Research Framework, identified gaps, and suggested additions to ensure both conceptual rigor and practical feasibility. This process was crucial to establishing a clear theoretical foundation and mapping indicators to the framework, thereby supporting the content validity of the measure under development [3,4].

In the first round of the Delphi process, seven out of ten expert panelists remotely reviewed the initial set of ecological-level indicators. The panelists also suggested additional indicators and provided justifications for their inclusion. Based on their responses, the research team categorized indicators as retained, to be discussed, or discarded. Indicators were retained if at least four panelists rated them as strongly or very important, flagged for discussion if at least three panelists rated them as moderately or somewhat important, and discarded if four or more panelists rated them as unimportant. In the second round, the full panel of ten experts met in person with the content development team and the measurement team to review and discuss the results from Round 1. During the discussion, panelists anonymously re-voted on each indicator using the Group Wisdom platform, which aggregated responses to reveal consensus and disagreement. Finally, in the third round, seven panelists worked offline to finalize the set of ecological-level indicators and select appropriate data sources and measures for each. They rated each indicator’s relevance to structural racism, assessed whether it fit within the appropriate domain of the NIMHD Research Framework, and evaluated its feasibility for ecological-level measurement. The panel excluded any indicator if more than half of the participants recommended its removal. For indicators with multiple data source options, they selected the preferred source if at least 50% of the panelists agreed. This final round ensured the selected indicators were not only conceptually grounded but also practically feasible for inclusion in the structural racism index.

Through this process, we identified a set of indicators that captured key dimensions of structural racism to serve as the foundation for developing an index of racial inequities. These indicators spanned multiple domains, including access to credit, housing discrimination, educational attainment, employment disparities, and inequities in healthcare access. Together, they reflect the multifaceted and systemic nature of structural racism across critical social and institutional systems.

### 2.4. Five-Step Approach for Data Extraction

Through the Delphi process, 144 ecological-level structural racism indicators were identified, providing a comprehensive framework for the development of the ecological-level index. To ensure the validity and appropriateness of these indicators for creating the index, it was essential to evaluate them using empirical data. As a result, we developed a de novo 5-step approach to guide the process of gathering and evaluating relevant data from various sources, including governmental agencies such as the U.S. Census Bureau, Bureau of Economic Analysis, Bureau of Transportation Statistics, Federal Bureau of Investigation, Bureau of Justice Statistics, and the CDC National Center for Health Statistics.

Phase II of Figure 2 describes the 5-step process followed by the research team to ensure accuracy, relevance, and comprehensiveness of the data used to measure the ecological-level indicators. The process involved data search, variable identification, data storage, cross-review, and final evaluation. The first four steps were executed by five team members, including two postdoctoral researchers and three doctoral students. The final step was carried out by the measurement and the content development team.

Step 1: Data search

The first step in the process was to conduct a search to identify datasets that could accurately measure the ecological-level indicators of structural racism. Three criteria were established to guide the search and ensure that the datasets met the necessary standards for the ecological-level index. These included (a) relevance of datasets, (b) geographic level data, and (c) comprehensive data coverage.

- Relevance of datasets: The datasets had to be relevant to the ecological-level indicators identified during the Delphi process. Each dataset needed to contain variables that directly measured the intended indicators of structural racism. For example, to measure an indicator related to housing discrimination, the dataset would need to include data on mortgage denial rates, homeownership rates, or rental denial rates based on race or ethnicity. Relevance also required datasets to be both recent and part of an ongoing or regularly updated data collection effort. This ensured the ability to monitor changes over time and capture the dynamic nature of structural racism, rather than relying on outdated or static snapshots.
- Geographic level data: With the second criterion, the datasets needed to be disaggregated at the local or community level. Our preferred unit was the smallest unit of analysis possible, which is the ZIP code or county level. This granularity was critical for accurately capturing the localized impact of structural racism, as structural factors may differ significantly across geographic regions [21,22].
- Comprehensive data coverage: In order to ensure the results were generalizable and representative of the entire United States, the datasets had to cover over 80% of the country. Ideally, these datasets needed to represent more than 40 states to capture the national variation in structural racism and its impacts. This level of coverage ensured that the ecological-level index would reflect a broad and diverse set of geographical and demographic contexts. Once the datasets were identified, their respective URLs were cataloged for easy access by the team. This cataloging process allowed the research team to efficiently retrieve datasets during later stages of the project and track any updates or changes to the data over time.

Step 2: Variable identification

After relevant datasets were located, the next step was to identify the specific variables to construct the ecological-level indicators of structural racism. The team reviewed each dataset to find variables closely linked to the ecological-level indicators. For example, educational inequities led to a focus on variables such as graduation rates and number of school suspensions, while economic access involved considering income inequality, unemployment rates, and business loan access. Each variable was assessed for its relevance to the conceptual framework of structural racism, ensuring it captured the factors contributing to health disparities and social inequities. The team also considered the potential limitations of each variable, such as inconsistencies in data reporting.

Step 3: Data storage

Once appropriate variables were identified, the datasets were stored in an organized folder accessible to the entire research team. This ensured easy access to data and prevented loss or disorganization. The folder was structured by indicator and dataset source for easy retrieval. Any issues during the download or storage process, such as access or file format problems, were documented to track and resolve potential roadblocks promptly. To address data security concerns, only secondary datasets that were publicly available and did not contain personally identifiable information were used.

Step 4: Cross-review

The fourth step in the process was cross-review, where the selected datasets and variables were thoroughly reviewed for accuracy and alignment with the intended ecological-level indicators. This step was critical for ensuring that the data used in the ecological-level index were reliable and valid. During cross-validation, the research team conducted a thorough examination of each dataset to verify that the variables accurately reflected the ecological-level indicators of structural racism. To ensure rigor in the review process, each team member cross-checked the work of two other peers. This peer-review approach involved checking for consistency across datasets, verifying that the variables were measuring the intended concepts, and ensuring that the data were up-to-date and correctly reported. The team also critically examined each dataset for potential sources of bias, including underrepresentation of specific racial or ethnic populations and geographic areas. Any discrepancies, errors, or concerns identified during this phase were resolved collaboratively before advancing to the next stage of index development.

Step 5: Final evaluation

The final step in the data preparation process was variable evaluation, which was carried out by both the content development and measurement teams. The team assessed whether the selected variables and their corresponding datasets were appropriate for inclusion in the ecological-level index. They evaluated the variables based on the following criteria:

- Variable relevance: The content development team examined each selected variable for its conceptual alignment with the indicators of structural racism. They determined whether the variable effectively captured an aspect of systemic inequality (such as segregation, access to resources, or exposure to institutional discrimination) and whether it was closely tied to the lived experiences and outcomes of marginalized populations. Two questions guided the assessment of the relevance of each identified variable: (1) Does the variable accurately measure the ecological indicator it was intended to represent? and (2) Is the variable directly related to the concept of structural racism and its impact on marginalized populations? Negative responses to both questions led to the exclusion of variables.
- Data completeness: The utility of a variable in an ecological-level index depends in part on its coverage. As a result, the research team confirmed whether data for each variable were available across different geographic units (e.g., zip codes, counties, or states) as well as population subgroups, especially Blacks and Hispanics/Latinos. Variables with substantial data missingness, limited geographic representation, or underreporting among Black and Hispanic/Latino populations posed a risk to the robustness of the index and were subject to further scrutiny. To determine completeness, we answered two questions: (1) Does the variable have sufficient coverage across geographic areas and for Black and Hispanic/Latino population groups? and (2) Are there significant gaps or missing data that would undermine the index’s comprehensiveness? Variables were eliminated based on negative responses to both questions. Sufficient coverage was defined as the availability of data for both Black and Hispanic/Latino populations across the majority of geographic units included in the dataset (typically ≥80% of eligible ZIP codes, counties, or states), with stable estimates that were not suppressed or masked due to small cell sizes. Variables that only captured Black–White disparities or lacked disaggregated data for Hispanic/Latino populations across most geographic units were excluded.
- Consistency: In evaluating consistency, we focused on the uniformity of variable definitions, collection methods, and reporting practices across datasets. Variations in how variables were defined, such as differences in race categories, income thresholds, or measurement units, posed risks to the reliability of the index. We reviewed metadata and documentation to identify discrepancies in data collection timeframes, methodologies, and coding practices. Depending on the degree of inconsistency, variables were either flagged for potential harmonization at a later stage or excluded entirely to preserve methodological rigor.

Each variable was carefully evaluated to ensure that it met these criteria and was suitable for inclusion in the final ecological-level index. When variables were found to be insufficient or problematic due to poor data quality, limited relevance, or inconsistent reporting, they were excluded from the final ecological-level index. In such cases, we sought alternative variables that could serve as more effective proxies. This iterative, criteria-driven approach helped to safeguard the integrity of the ecological-level index and ensured that the final set of variables was both theoretically grounded and empirically sound.

At this stage, the index is being constructed from the selected ecological-level indicators spanning multiple domains of structural racism. While the final weighting and aggregation method is still under consideration, options include computing a composite score from the average of geographic-level percentiles across variables or applying dimension-reduction techniques such as principal component or factor analysis. More complex approaches, including multilevel modeling, may also be explored to account for hierarchical geographic effects. Details on the completed multilevel, multidimensional index will be reported in future publications.

## 3. Results

Developing an index to measure structural racism at the ecological level presents a range of methodological challenges, stemming from both the complexity of the construct itself and the limitations of available data. Translating this multifaceted phenomenon into measurable indicators requires careful navigation of conceptual, technical, and practical issues. The challenges encountered in applying the 5-step approach described above were significant and shaped each stage of the process. In the sections that follow, we describe the challenges and outline the strategies we employed to address them. Table 1 presents a summary of the key challenges we encountered and their resolutions.

### 3.1. Limited Geographic Data Challenge

One of the primary data-related challenges was the limited availability of granular geographic data, which led to a mismatch between the intended geographic levels for measuring structural racism indicators and the levels at which data were available. For instance, while the research aimed to utilize detailed data at the ZIP code or county level, many relevant datasets such as the FBI’s Uniform Crime Reporting (UCR) program, the U.S. Census Bureau’s Annual Business Survey (ABS) and the U.S. Department of Health and Human Services (HHS) Child Welfare Outcomes Report Data were only accessible at the state level. This geographic mismatch limited the ability to capture ecological-level variations in structural racism across regions, thereby reducing the depth and specificity of the analysis. Additionally, acquiring more granular data sometimes involved high costs, which posed budgetary constraints. To address this challenge, we employed proxies and supplemental data sources to fill the gaps. When granular data at the ZIP code or county level was unavailable, we used alternative sources such as state-level estimates with more detailed geographic breakdowns. This allowed for a more accurate reflection of ecological-level conditions across states, despite the limitations of the original datasets.

### 3.2. Inadequate Data Description

Vague or insufficient dataset descriptions posed a barrier to identifying appropriate indicators of structural racism. Many data sources lacked clear explanations of variable definitions, data collection methods, or geographic scope, making it difficult to determine their relevance or reliability. To address this issue, we conducted a thorough review of available data documentation and consulted supplementary literature to better understand the context, limitations, and structure of each dataset. This additional investigation was essential for accurately interpreting variables and ensuring that selected indicators aligned with the conceptual framework guiding the index.

### 3.3. Variations in Data Quality Challenge

Variations in data quality posed a significant challenge throughout the research. Some sources contained more complete, high-quality data, while others were incomplete or less useful, introducing potential bias into the analysis. For example, the FBI’s Uniform Crime Reporting (UCR) Program relies on data voluntarily reported by law enforcement agencies, and participation can vary, with some smaller or rural jurisdictions either failing to report consistently or not reporting at all, leading to potential underreporting. Additionally, inconsistent operational definitions and coding schemes across datasets further compounded the issue. Different sources defined key variables in slightly different ways or used varying categories and codes to represent the same concept. These discrepancies in how data were measured, categorized, and coded create difficulties in aligning the datasets for analysis and could potentially undermine the validity of the index. To address the challenges posed by variations in data quality, we prioritized datasets from well-established and reliable sources. We focused on selecting sources that used similar methodologies and offered thorough documentation to reduce discrepancies across datasets. In particular, we frequently relied on data from the American Community Survey (ACS), collected by the U.S. Census Bureau, as well as data from other reputable government agencies known for their standardized collection practices and transparent methodologies [23,24].

### 3.4. Reporting Frequency Challenge

Another critical challenge was reporting frequency. Data sources often reported information at varying temporal intervals, which made it difficult to track trends over time. For instance, employment data from the Bureau of Labor Statistics are released monthly, crime data from the FBI’s Uniform Crime Reporting (UCR) Program are released annually, while Health data from the CDC’s National Health and Nutrition Examination Survey (NHANES) and data on housing conditions, characteristics, and affordability from the American Housing Survey (AHS) are reported biennially. In the case of the American Community Survey (ACS), the U.S. Census Bureau reports data on varying timelines, depending on the geographic area and the type of estimates. For larger areas, such as states or large metropolitan regions, data are reported annually. However, for smaller geographic areas (like small towns or specific neighborhoods), the ACS provides five-year estimates, which combine data collected over a five-year period to ensure statistical reliability. The annual data are made available the following year, while the five-year estimates are typically released in the third year after the end of the five-year collection period. These differences in reporting frequency create challenges in obtaining up-to-date information, especially for local or rapidly changing populations. As a resolution to this challenge, we developed aggregation methods that aligned temporal data. We standardized the frequency of data reporting by aggregating data points to a common temporal scale, such as using five-year estimates of annually reported data or one year estimate of monthly reported data, depending on the needs of the analysis. These adjustments ensured that datasets could be compared over time and trends could be consistently tracked.

### 3.5. Data Adaptability Challenge

Another significant challenge was the lack of adaptability in publicly available datasets for measuring structural racism. Many datasets did not contain the specific variables required to capture key dimensions of structural racism, such as indicators related to affirmative action, housing discrimination, criminal justice disparities, or education inequality. As a result, the research team had to either seek out alternative data sources or repurpose existing, broader variables as proxies. To address this challenge, we explored alternative sources, including government reports and specialized datasets that were not immediately obvious. In cases where no direct measures were available, closely related variables were adapted to approximate broader structural racism indicators, such as using violent encounters with the police and the number of police stops by race as a proxy for police bias. These adaptations ensured that key aspects of structural racism were still incorporated into the analysis, even when the exact desired data were not available.

### 3.6. Limited Use of AI Tools Challenge

Another challenge encountered was the limited effectiveness of AI tools in the dataset selection process. Although AI-assisted tools could generate suggestions for potentially relevant datasets [25], these recommendations were often overly broad, tangential, or misaligned with the specific research objectives. As a result, significant manual effort was required to evaluate and validate the relevance and quality of the AI-suggested datasets. We treated AI-generated suggestions as preliminary leads rather than definitive sources. Each suggestion was manually reviewed to assess its relevance, accuracy, and alignment with the research objectives. This validation process included quality checks to ensure that datasets met the specific criteria required for measuring structural racism. By integrating human oversight with AI support, we were able to streamline dataset identification while maintaining the rigor and specificity necessary for the analysis.

## 4. Discussion

The methodological challenges summarized here highlight the challenges in developing a structural racism measure. Given the multifaceted nature of structural racism [1,11], constructing a reliable and meaningful index demands thoughtful decisions on how to manage imperfect indicators while still reflecting the dimensions of structural racism. For future researchers who are interested in creating indices to measure similarly complex social constructs, several overarching practical considerations and strategies are crucial to ensure the final product is valid, reliable, and reflective of the nuances of the issue being studied. The strategies we used to navigate our challenges offer practical insights on how we developed our ecological-level index and insights that are applicable to similar research efforts.

One of the first hurdles we encountered in ecological-level index construction was selecting the right data. Each data source has strengths and weaknesses [26,27] and we prioritized data that was comparable across time and geography [28]. When building similar indices, researchers should plan for time to find alternative data sources and harmonize data so that indicators are comparable [21,28]. Data that is available for ecological level indices often varies by granularity, coding of variables, and variable definitions and given that ecological measures will inevitably require multiple data sources, creation of other ecological indices must address these issues to avoid inconsistencies in the final ecological-level index. Researchers should also be careful about simply collapsing variable categories and geographies, as these differences could be critical for accurately capturing their construct of interest. They should be prepared to devote time and resources to this step, as proper alignment of variables is essential for constructing an accurate and meaningful ecological-level index.

A particularly salient challenge in this work was developing a structural racism index applicable to Hispanic/Latino populations, given the distinct ways structural racism manifests for this group relative to Black populations. Hispanic/Latino communities experience systemic inequities shaped by language-based discrimination, immigration enforcement policies, and restricted access to culturally relevant health and social services. These unique structural processes necessitate careful selection of ecological-level indicators that are both relevant to Hispanic/Latino experiences and compatible with indicators used for Black populations, allowing for comparability across groups. However, many publicly available datasets historically focus on Black-White disparities, limiting the granularity and geographic coverage needed to capture inequities affecting Hispanic/Latino communities. These conceptual and data-related challenges underscore the importance of complementary individual-level measures to capture lived experiences and interactions with structural forces. By addressing both ecological- and individual-level dimensions, our approach seeks to provide a more comprehensive and inclusive framework for measuring structural racism across multiple populations, highlighting the methodological and theoretical considerations needed when extending measurement beyond traditional Black-White comparisons.

Our experience also reinforces the need for adaptability throughout the process of building an ecological-level index. Researchers should be prepared to adjust their methodology when data reflecting each indicator have significant shortcomings. Indices, data sources, decisions and the ecological index’s structure should be regularly re-evaluated in an iterative process. The re-evaluation process should also include checks that each component of the index is aligned with the research goals and accurately reflects the issue being studied. The first version of an ecological-level index should be considered a foundation for further refinement. Similar to the measure development process, our work supports the view that refinement and continuous improvement are key to creating a rigorous ecological level index. Building flexibility into the methodology of calculating the index will allow the measure to evolve over time.

In addition, transparency in data selection and methodological decisions is especially critical given the substantial variations in data quality, completeness, and accessibility. We documented each step of our ecological-level index construction process, from data selection to variable harmonization and methodological choices. By doing so, we ensured that the final index is reproducible, enables critical assessment by other researchers, and fosters trust in the results. Furthermore, clearly outlining how we addressed missing data, geographic variations, and measurement inconsistencies enhances the credibility of the index and supports its effective use by researchers in the field. Comprehensive documentation of the entire process also facilitates future improvements and adaptations, making it easier for other researchers to replicate or refine our work.

To ensure that the ecological-level index accurately captures structural racism and is suitable for research and policy applications, rigorous validation is essential. Our planned validation approach includes several complementary strategies. First, we will assess internal consistency to ensure the indicators coherently reflect the underlying domains of structural racism. Second, we will evaluate external validity by comparing the index against existing measures of structural racism and related social determinants of health, as well as testing its predictive validity for relevant health and social outcomes. Third, we will examine geographic and temporal stability, assessing whether the index produces consistent patterns across different regions and time periods. Finally, we will incorporate participatory validation, engaging community members, practitioners, and subject-matter experts to evaluate whether the index reflects lived experiences and captures relevant structural processes. By integrating these approaches, we aim to provide a robust, transparent, and credible measure that is both scientifically rigorous and meaningful to the communities it represents.

### 4.1. Implications for Future Research

We are in the process of developing methodological guidelines to support future researchers who aim to construct similar ecological indices of structural racism. These guidelines will provide directions on how to statistically address the data-related challenges outlined in this study, including approaches for handling missing and unevenly distributed data, selecting and justifying indicator weighting and aggregation strategies, evaluating alternative index structures (e.g., additive versus dimension-reduction approaches), and integrating ecological- and individual-level data using multilevel modeling frameworks. Many of these considerations are illustrated in the present paper through our handling of variable harmonization, cross-review procedures, and discussions of data granularity, validation, and multilevel extensions.

To ensure methodological rigor, future researchers should systematically document each stage of the index development process from data identification and variable harmonization to analytic modeling and validation decisions. Such documentation enhances reproducibility, facilitates critical peer assessment, and fosters trust in the resulting index. Moreover, explicit reporting of how issues such as missing data, geographic disparities, and measurement inconsistencies are addressed strengthens the credibility and practical utility of ecological-level indices. Comprehensive documentation also enables future scholars to replicate, refine, or extend existing indices, thereby contributing to the cumulative growth of evidence in this field.

Given the challenges of secondary data adaptability, we recommend that once an ecological-level index is constructed, it must undergo rigorous validation to ensure it accurately reflects the phenomenon being measured [29]. Validation should include both internal consistency checks and external comparisons with existing measures or related empirical findings. This dual approach helps verify reliability and construct validity. Additionally, validation should not occur in isolation. Engaging with the broader research community, practitioners, subject-matter experts, and particularly community members most affected by structural racism can provide critical feedback on the robustness, interpretability, and real-world applicability of the index. Such participatory engagement not only enhances scientific credibility but also ensures that the resulting indices remain grounded in lived experiences and are responsive to the realities of structural inequity.

### 4.2. Limitations

It is important to point out that using ZIP code-, county-, and state-level data to construct a structural racism index presents some limitations. These geographic units are administrative and may not always align with sociologically meaningful boundaries. Aggregation at these levels can mask local disparities, particularly when diverse communities are averaged together [30,31]. Although state-level data often capture meaningful policy environments that shape daily lived experiences, including variation in criminal justice, education, and social welfare systems, state-level aggregation may be too broad for certain indicators of structural racism. For example, measures of residential segregation, school disciplinary practices, or access to health-promoting resources can vary substantially within states across counties or ZIP codes, and state-level estimates may obscure these localized inequities. In such cases, finer geographic resolution is necessary to adequately capture place-based manifestations of structural racism

Relatedly, data availability and quality vary by geographic level [26]. Many key indicators, such as incarceration rates or school segregation, are inconsistently reported or unavailable at finer scales like ZIP codes, especially for smaller racial and ethnic populations such as Blacks and Hispanics/Latinos. This can lead to unstable estimates in rural or underrepresented areas. These limitations heighten the risk of ecological fallacy, where area-level patterns are incorrectly assumed to reflect individual-level experiences. To mitigate this risk, the ecological-level structural racism index is intended to be used within a multilevel analytic framework in which individuals are nested within geographic contexts. In such models, the index functions as a contextual exposure, while individual-level measures capture personal experiences and characteristics, allowing for the examination of cross-level interactions and effect modification. This approach enables researchers to assess how structural conditions shape individual outcomes while avoiding assumptions that area-level associations directly reflect individual-level processes. Researchers must therefore approach geographic data with caution, prioritizing reliable sources, conducting sensitivity analyses, and considering multi-level approaches where feasible [26,32,33,34,35,36].

An additional limitation concerns the sensitivity of publicly available data used to construct ecological-level measures of structural racism. Although aggregate geographic data minimize risks related to individual identification, the use of administrative and population-level datasets must be considered within the historical context of data misuse, surveillance, and the erosion of agency among minoritized populations. These histories have contributed to persistent community mistrust regarding how data are collected, interpreted, and applied, particularly when used to inform policy or resource allocation. While our study relies exclusively on secondary, publicly available data, these ethical considerations remain salient, especially as structural racism measurement advances toward finer geographic resolution and the integration of individual-level data. Future work, including the development of complementary individual-level measures, should prioritize ethical reflexivity, transparency, and community engagement to ensure that measurement efforts are responsive to community concerns and do not inadvertently reproduce harm.

## 5. Conclusions

Building an ecological-level index for structural racism requires navigating a landscape of fragmented, uneven, and imperfect data. The methodological challenges, ranging from geographic and temporal misalignments to limited data relevance and accessibility, reflect broader epistemological constraints in the measurement of systemic inequities. Addressing these challenges necessitates both technical innovation and critical flexibility. Integrating multiple data sources, harmonizing temporal trends, adapting proxy indicators, and exercising informed human judgment in AI-assisted suggestions collectively enhanced the validity and interpretability of our ecological-level index. Future work should focus on data infrastructure reform and cross-sector collaborations to generate standardized, accessible, and theoretically grounded indicators capable of capturing the dynamic and place-based dimensions of structural racism.

To reduce the risk of ecological fallacy, where area-level patterns are incorrectly assumed to reflect individual-level experiences, we are developing an individual-level measure of structural racism. This component is being designed as a survey-based instrument intended to capture experiential dimensions of structural racism, which will complement the ecological-level index, enabling a multilevel, multidimensional approach to capturing structural racism. By integrating both individual and contextual data, we aim to more accurately reflect the lived realities of Black and Hispanic/Latino populations and provide a more nuanced understanding of how structural racism operates across different levels of society.

## Figures and Tables

**Table 1:** Data challenges and their potential impacts on the validity of an ecological-level structural racism index.

Challenges	Description	Potential Impacts	Resolutions
Limited Geographic Data	Misalignment between structural racism indicators and available dataset aggregation levels (e.g., state-level vs. county level).	May obscure localized inequities and reduce sensitivity to place-based manifestations of structural racism.	Used proxies and supplemental sources to approximate ecological-level granularity when county or ZIP code data were unavailable.
Inadequate Data Description	Vague dataset descriptions hindered indicator identification.	Increases the risk of construct misclassification and threatens content validity if variables do not accurately reflect intended indicators.	Reviewed data documentation and supplementary literature for a better understanding.
Variations in Data Quality	Variations in data quality introduced potential bias and complicated efforts to align datasets for analysis.	May reduce reliability and comparability across indicators and geographic units.	Prioritized similar data sources to minimize variability and interpreted measures cautiously.
Reporting Frequency	Temporal mismatches in data reporting complicated analysis.	Limits temporal comparability and may affect the stability of the index over time.	Developed aggregation methods to align temporal data.
Data Adaptability	Publicly available datasets often lacked variables relevant to structural racism.	Constrains construct validity by requiring the use of proxy indicators that may only partially capture structural processes.	Explored alternative sources and adapted broad indicators to approximate variables of interest.
Limited Use of AI Tools	AI tools provided broad but sometimes irrelevant dataset suggestions, requiring manual validation.	Minimal direct impact on validity but increased risk of misalignment without careful human review.	Used AI-generated suggestions as starting points, followed by manual evaluation, quality checks, and alignment with research goals.

## Data Availability

Data sharing is not applicable to this article, as no new data were generated and the article describes methodological processes and challenges in creating an ecological-level index.

## Abbreviations

The following abbreviations are used in this manuscript:

NIMHD	National Institute on Minority Health and Health Disparities
US	United States
CDC	Centers for Disease Control and Prevention
AI	Artificial Intelligence
ZIP	Zone Improvement Plan
